# Leaf Transcriptome Sequencing for Identifying Genic-SSR Markers and SNP Heterozygosity in Crossbred Mango Variety ‘Amrapali’ (*Mangifera indica* L.)

**DOI:** 10.1371/journal.pone.0164325

**Published:** 2016-10-13

**Authors:** Ajay Kumar Mahato, Nimisha Sharma, Akshay Singh, Manish Srivastav, Jaiprakash, Sanjay Kumar Singh, Anand Kumar Singh, Tilak Raj Sharma, Nagendra Kumar Singh

**Affiliations:** 1 ICAR-National Research Centre on Plant Biotechnology, Pusa Campus, New Delhi, India; 2 The Division of Fruits and Horticultural Technology, ICAR-Indian Agricultural Research Institute, Pusa, New Delhi, India; National Institute for Plant Genome Research, INDIA

## Abstract

Mango (*Mangifera indica* L.) is called “king of fruits” due to its sweetness, richness of taste, diversity, large production volume and a variety of end usage. Despite its huge economic importance genomic resources in mango are scarce and genetics of useful horticultural traits are poorly understood. Here we generated deep coverage leaf RNA sequence data for mango parental varieties ‘Neelam’, ‘Dashehari’ and their hybrid ‘Amrapali’ using next generation sequencing technologies. De-novo sequence assembly generated 27,528, 20,771 and 35,182 transcripts for the three genotypes, respectively. The transcripts were further assembled into a non-redundant set of 70,057 unigenes that were used for SSR and SNP identification and annotation. Total 5,465 SSR loci were identified in 4,912 unigenes with 288 type I SSR (n ≥ 20 bp). One hundred type I SSR markers were randomly selected of which 43 yielded PCR amplicons of expected size in the first round of validation and were designated as validated genic-SSR markers. Further, 22,306 SNPs were identified by aligning high quality sequence reads of the three mango varieties to the reference unigene set, revealing significantly enhanced SNP heterozygosity in the hybrid Amrapali. The present study on leaf RNA sequencing of mango varieties and their hybrid provides useful genomic resource for genetic improvement of mango.

## Introduction

Mango (*Mangifera indica* L.) is an evergreen dicotyledonous angiosperm. Although several tetraploid *Mangifera* species are reported, cultivated mango is a diploid tree (2n = 40) with a relatively small genome size of 439 Mbp [1, 2]. The genus *Mangifera* belongs to order Sapindales of the family Anacardiaceae [1]. The major mango producing countries are India, China, Thailand, Indonesia and Pakistan. India is the largest producer of mango in the world, with an annual production of 18–19 Mt from an area of 2.31 Mha, contributing about 40% of the total world production (FAOSTAT-2014 [3]). It is grown in almost all the states of India, Andhra Pradesh tops in total production, whereas Uttar Pradesh tops in area. Andhra Pradesh, Uttar Pradesh, Bihar, Karnataka, Maharashtra, West Bengal and Gujarat together contribute about 82% of the total mango production in India [4]. More than 1,000 varieties of mango exist in India today, which contribute 39.5% of the total fruit production in the country [5–6]. There are 25 major commercial cultivars of mango some of which are highly preferred in the International market. According to Agricultural and Processed Food Products Export Development Authority (APEDA) India has exported 41,280 tons of mango worth around 50.7 million US dollars during 2013–14, United Arab Emirates, United Kingdom, Saudi Arabia, Kuwait, Qatar and United States are the major exporting destination. Mango is used in various forms such as pickle, chutney, jelly, cool summer drinks and as vegetable dishes. Peel and pulp of mango are rich in carotenoids and polyphenols like xanthonoid and mangiferin [7, 8]. Notwithstanding its enormous benefits, mango is a difficult plant to handle in breeding due to long juvenile phase, high heterozygosity, heavy fruit drop and large area required for assessment of the hybrids [9–12]. Cultivars from North India have the problem of alternate bearing, while South Indian cultivars are generally regular bearer. ‘Neelam’ is a high yielding, late season mango variety of South India that has regular bearing, medium size fruits, good flavor, yellow fibreless soft flesh and good keeping quality. ‘Dashehari’ a mid-season and most popular varieties of North India, with medium fruit size, sweet, pleasant flavor, thin stone, firm and fibreless pulp, and good keeping quality but it has the problem of alternate bearing [9]. A cross between ‘Dashehari’ and ‘Neelam’ resulted in the development of ‘Amrapali’, a popular dwarf regular bearing variety with small fruit size, good taste and keeping quality. Molecular breeding and gene discovery in mango has been limited by paucity of informative molecular markers, which is primarily due to non-availability of the mango genome sequence and other genomic resources. A variety of molecular markers including AFLP, RAPD, RFLP, COSII and SSR have been developed and applied to intra and inter-specific crosses of mango but with limited success in molecular breeding [13–17]. Among different types of molecular markers that have been developed during the past three decades, SSR and SNP are highly informative markers [18, 19]. Microsatellite or SSR markers are one of the most informative and versatile DNA-based markers used in plant genetic research [19, 20], but traditionally their development has been costly and difficult. Prior to the arrival of next generation sequencing (NGS) technologies, Low throughput electrophoresis or capillary sequencing was used for SNP discovery [21, 22]. Using NGS technologies and recent bioinformatics tools identification of SNP and SSR markers in a genome has become feasible and affordable [23, 24]. It allows efficient identification of large numbers of microsatellites at a lesser cost and effort as compare to the traditional approaches [21, 25, 26]. The main advantage of developing SSR markers from NGS transcriptome sequences is the increased possibility of finding associations with functional genes and therefore with phenotypes. Microsatellites in the coding region of genes may actually regulate gene expression and function, making them a valuable resource for genetic studies and breeding applications [27]. NGS technologies provide ultra-fast and inexpensive methods for unraveling the genome and transcriptome of plants [28]. These sequencing technologies can now be used for allele mining, gene discovery and genome-wide identification of SNP in non-model organisms [29–32]. The present study on sequencing of RNA from the leaves of mango varieties ‘Neelam’, ‘Dashehari’ and ‘Amrapali’ was aimed at identification of genic SSR and SNP loci and level of heterozygosity in the genomes of these selected mango varieties and their hybrid.

## Materials and Methods

### Plant material

Leaf samples of three mango genotypes ‘Neelam’, ‘Dashehari’ and ‘Amrapali’ were taken from the orchard of the Division of Fruits and Horticultural Technology, IARI, New Delhi, India. The fresh leaves were immediately frozen in liquid nitrogen and stored at −80°C until RNA extraction. For SSR validation we have used fresh leaf samples of 8 different popular varieties of mango *viz*., ‘Neelam’, ‘Dashehari’, ‘Amrapali’, ‘Chausa’, ‘Pusa Lalima’, ‘Ratual’, ‘Mallika’ and ‘Alphonso’.

### RNA isolation and library preparation for sequencing

Total RNA was extracted from the leaves using Purelink miRNA isolation kit according to manufacturer’s instructions (Invitrogen). Quantification of RNA was done using NanoDrop spectrophotometer and quality check for DNA contamination was done by electrophoresis in 1% denaturing agarose gel. To assess RNA integrity the samples were run on RNA 6000 Pico chip Bioanalyzer (Agilent). The transcriptome library was prepared after quantification and quality check of the Poly(A) RNA using SOLiD total RNA Seq kit for ‘Neelam’ and ‘Dashehari’ and Illumina MiSeq for ‘Amrapali’, respectively.

### *De novo* transcript assembly

High quality sequence reads of cDNA libraries were used to generate transcript shotgun assembly (TSA) contigs. Transcripts for ‘Neelam’ and ‘Dashehari’ were assembled via *de novo* assembly approach using Velvet-Oasis software, which have been developed for short read assembly of transcriptome data and are based on de-Brujin graph algorithm [33, 34] Longer sequence reads of ‘Amrapali’ were assembled using CLCGenomics Workbench (version 6.5.1) [35]. The assembled TSA contigs of the parents and hybrid were further merged in to a non-redundant set of unigene contigs using CAP3 [36] with default parameters (overlap length cutoff = 40bp and overlap percent identity cutoff = 90%). This unigene set was used for mining of SSRs and for a three way alignment of sequence reads for SNP identification among the three mango genotypes.

### SSR detection and primer designing

The final assembled unigene contigs were used for mining of genomic-SSRs using MISA [37] and SSR specific primers were designed using BatchPrimer3 V1.0 [38]. The SSR loci containing repeat units of 2–6 nucleotides only were considered. The criteria for minimum SSR length were defined as 6 reiterations for di-nucleotide SSR and 5 reiterations for tri-, tetra-, penta- and hexa-nucleotide SSRs, mono-nucleotide repeats and complex SSR were excluded [39]. The parameters for designing of primers from SSR flanking sequence were: primer lengths = 20–25 bp; PCR product size = 100–250 bp; annealing temperature = 65°C; GC content = 40–60% with an optimum of 50%; only single consecutive bases of Gs and Cs at the 3’ end of both primers were specified. Remaining parameters were kept at the default setting for BatchPrimer3 V1.0.

### SSR marker validation

Genomic DNA was isolated from leaf samples of eight genotypes using CTAB method [40], quantified by UV260 absorbance and adjusted to a final concentration of 30 ng/μl. A set of 100 genic-SSR markers with SSR lengths of 20 bp or above (Type I SSR) were tested for amplification using genomic DNA of ‘Amrapali’ for optimization of the annealing temperature. The PCR reactions were performed in a BioRad Thermal Cycler. Each PCR reaction consisted of 1.5 μl of 10X reaction buffer, 0.20 μl of 10 m MdNTPs (133 μM), 1.5 μl each of forward and reverse primers (10 pMol), and 2.5 μl of template genomic DNA (75 ng), 0.15 μl of *Taq* DNA polymerase (0.75 U) in a final reaction volume of 15 μl. The PCR reaction profile was denaturation at 94°C for 5 min followed by 35 cycles of 94°C for 1 min, 55°C for 1 min, 72°C for 1 min and finally, 72°C for a final extension of 7 min. Re-screening of primers that did not amplify at these conditions was done by decreasing the annealing temperature sequentially by 1°C, and for the primers producing multiple bands, by increasing the annealing temperature by 1°C [21]. The optimized SSR primers were then used for PCR amplification of multiple varieties of mango. The PCR products were separated by electrophoresis in 4% Metaphor agarose gels (Lonza, Rockland ME USA) containing 0.1 μg/ml ethidium bromide in 1X TBE buffer at 130 V for 4 h. After electrophoresis, PCR products were visualized and photographed using a gel documentation system Fluorchem^™^ 5500 (Alfa Innotech Crop., USA). The SSR profiles were scored manually, each allele was scored as present (1) or absent (0) for each of the SSR loci. The SSR markers giving consistent expected size products only (100–250 bp) were used for further analysis of variation.

### Annotation and functional classification of the unigene TSA contigs

For the annotation of TSA unigenes, BLAST algorithm [41] was used to search for similarity against a locally configured non-redundant (nr) protein database of NCBI (as on 06 December, 2015) using BLASTX program [42] with cutoff E-value of ≤ 1e-6. The BLASTX result was saved in the.xml format and was imported into Blast2GO software [43] to assign Gene Ontology (GO) terms to the annotated unigene. Blast2GO classified unigenes under three GO terms called cellular component, biological process and molecular function. The GO annotated unigenes with GO terms were exported from Blast2GO in WEGO native format, and online tool WEGO (Web Gene Ontology Annotation Plot) [44] was used for the categorization of annotated unigenes in to three GO categories. For categorization of unigenes into 58 transcription factor families, unigenes were searched against the downloaded protein sequences of Plant Transcription Factor database version 3.0 (PlantTFDB 3.0) [45] using BLASTX with E-value cutoff of ≤1e-6. A COG classification was also performed using the same BLASTX search parameters against NCBI COG databases [46].

### Pathway mapping using KAAS

KEGG Automatic Annotation Server (KAAS) [47] was used for Pathway mapping and gene ortholog assignment of the unigenes. The KAAS gives functional annotation of genes by sequence similarity comparison against the manually curated KEGG GENES database [48]. Based on the similarity hits in the KEGG database using BLASTX (default threshold bit-score value of 60), unigenes were assigned with the unique enzyme commission (EC) numbers, and further mapped to the KEGG biochemical pathways.

### Multiple sequence alignment and SNP identification

The unigene set was used as reference for mapping of high quality filtered sequence reads from all the three varieties using BWA with default parameters [49]. To track and identify the variety specific reads mapping at a particular location we added the variety name at the end of header of each high quality reads using shell script. SAMtools software was used for conversion of aligned SAM file to BAM file and read sorting [50]. The SNPs were called using software VarScan version 2.7 [51] at highly stringent parameters: 1) minimum 10 reads mapped at each SNP position; 2) average base quality of ≥25; 3) minimum two reads for any SNP base call in each variety. Total mapped reads information including read name, base call with respect to reference position for all identified SNPs were fetched from the duplicate read removed BAM file using shell scripts and the final results were filtered and tabulated.

## Results and Discussion

### Functional categories of genes expressed in the mango leaves

A total of 60,359,815, 58,212,961 and 4,853,226 raw sequence reads were generated for ‘Neelam’ (mango_N), ‘Dashehari’ (mango_D) and ‘Amrapali’ (mango_A), respectively using two runs of SOLiD sequencing with average read length of 50 bp (mango_N and mango_D) and one run of Illumina Miseq 2x250 (mango_A) with average read length of 250 bp. After quality check, adapter trimming and removal of low quality reads, 53,617,132, 47,818,267 and 4,313,270 high quality reads were retained for ‘Neelam’, ‘Dashehari’ and ‘Amrapali’, respectively. We performed three separate de-novo assemblies using Velvet-Oases assembly pipeline and CLC Genomic workbench 6.5.1 for the SOLiD and Miseq data, respectively. The assembly resulted in 27,528, 20,771 and 35,182 transcripts for mango_N, mango_D and mango_A with transcript N50 values of 557 bp, 451 bp and 591 bp and largest transcript size of 3,129 bp, 2,958 bp and 5,891 bp, respectively (Table 1).

**Table 1:** NGS sequence and assembly statistics of mango leaf transcriptome varieties Neelam, Dashehari and their hybrid Amrapali.

Details	Neelam	Dashehari	Amrapali	N+D+A*
No. of raw reads	60,359,815	58,212,961	4,853,226	123,426,002
No. of high-quality reads (Q≥20)	58,617,132	57,518,267	4,313,270	120,448,669
No. of high-quality nucleotides (bp)	2,872,239,468	2,818,395,083	819,006,067	6,509,640,618
No. of assembled transcript/unigenes	27,528	20,771	35,182	70,057
Total length of assembled TSA (bp)	13,925,156	9,125,306	18,432,326	29,050,233
No. of transcripts ≥ 1000 bp	1,591	540	2,577	2,961
Longest TSA size (bp)	3,129	2,958	5,891	9,079
Average TSA size (bp)	506	439	524	415
N50 (bp)	557	451	591	470
GC%	42	43	41	42

*N+D+A = Assembly of transcripts from three varieties (Neelam + Dashehari + Amrapali).

**Fig 1:**
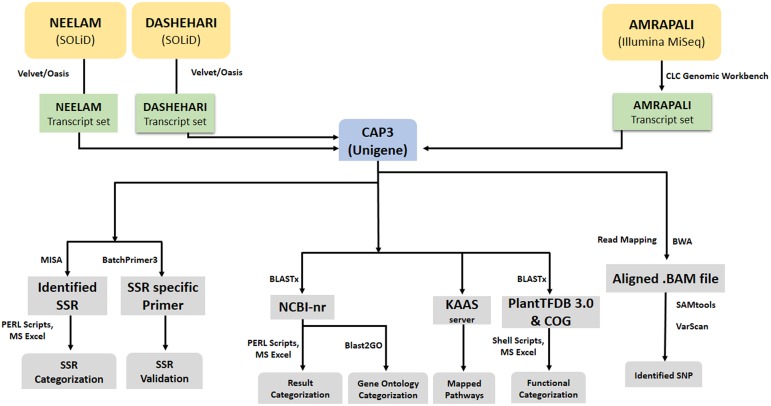
Flow diagram of mango transcriptome data analysis. Assembly, annotation and SSR/SNP identification in three varieties of mango (‘Neelam’, ‘Dashehari’ and ‘Amrapali’).

TSA contigs of individual genotypes were further assembled into 70,057 non-redundant unigenes set, which was used as reference for the identification of genic-SSR and SNP (Fig 1). The mean size of earlier reported 85,651 unigene contigs is 415 bp for the leaf transcriptome of mango variety ‘Langra’ with mean length of 238 bp [52], which is much lower than the present result. Further, sequencing of pooled transcriptome from pericarp and pulp of mango variety ‘Zill’ has resulted in 124,002 transcripts with average size of 838 bp [53]. Transcriptome from mango variety ‘Shelly’ generated 57,544 transcripts with an average length of 863 bp [54], and mesocarp transcriptome of mango variety ‘Kent’ is reported with 80,969 transcripts having mean length of 836 bp and N50 of 1,456 bp [55], which is significantly larger than our results as their transcriptome was sequenced using only Illumina platform which produces longer read length as compare to SOLiD sequenced reads.

Raw sequenced data described in this paper can be found in the Sequence Read Archive (SRA) database of the NCBI with SRA Accession number SRR1298995, SRR1297075, SRR1956775 under BioProject Number PRJNA193591, PRJNA193588, PRJNA279829 with TSA Accession number GBVX00000000, GBVW00000000, GEEEC00000000 for ‘Dashehari’, ‘Neelam’ and ‘Amrapali’, respectively.

For the functional annotation 70,057 unigene contigs were searched in the NCBI-nr protein database using BLASTx. As a result 39,798 (56.80%) of the unigenes showed significant similarity to known proteins and were functionally annotated while 30,259 (43.2%) unigenes showed no significant hits. Mango unigenes showed the highest similarity with *Citrus sinensis* (28.76%), followed by *Citrus clementina* (17.43%), *Theobroma cacao* (6.8%), *Jatropha curcas* (4.44%) and *Vitis vinifera* (4.19%) (S1 Fig). This result is consistent with the phylogenetic study of mango chloroplast DNA which reported *Citrus* to be most closely related to *Mangifera indica* [53].

**Fig 2:**
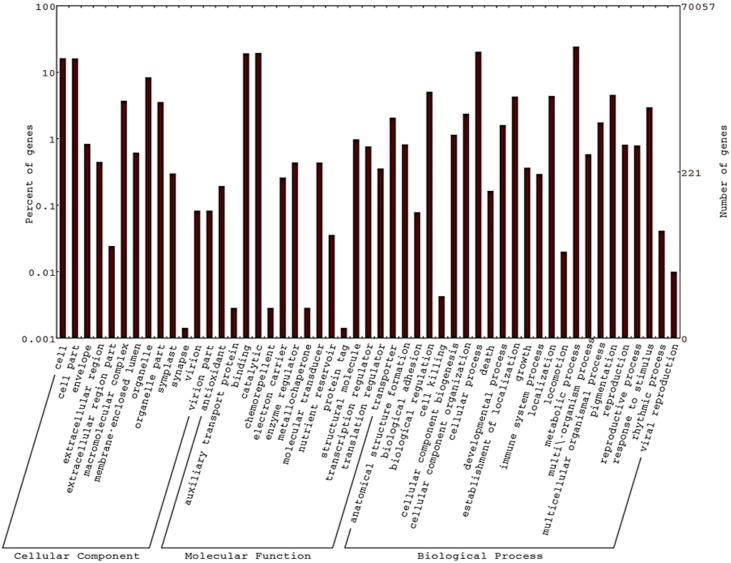
Gene Ontology (GO) classification of annotated mango leaf transcripts. Out of 70,057 transcripts, a total of 26,001 transcripts were classified into three main GO categories: Biological Processes, Cellular Component and Molecular Function.

Gene ontology (GO) terms were assigned successfully to 26,001 of the BLASTX annotated unigenes using BLAST2GO, which were broadly categorized into three main categories; biological process (BP), cellular component (CC) and molecular function (MF) and were further classified into 47 functional groups (Fig 2). The most abundant unigenes were in the biological process category followed by molecular function and cellular components. In the biological process category the highly represented GO terms were “metabolic process”, “cellular process” and “biological regulation” while in molecular function the highest represented GO terms were “catalytic activity” and “binding” whereas in cellular component category the highest represented GO terms were “cell”, “cell part” and “organelle”. Somewhat similar results have been reported earlier for ‘Langra’ and ‘Kent’ leaf transcriptomes with highly represented GO terms “cell” and “cell part” in the cellular component category, “metabolic process” and “cellular process” in biological process and “catalytic activity”, “binding” in molecular function categories [52, 55].

Total 8,958 unigenes were assigned Enzyme Commission (EC) numbers; the highly represented enzyme classes were hydrolases (3,335) transferases (3,091) and oxidoreductases (1,437). The large number of unigenes under these three major enzyme groups indicates expression of genes related to secondary metabolite biosynthesis pathway in the mango leaves [52, 53].

**Fig 3:**
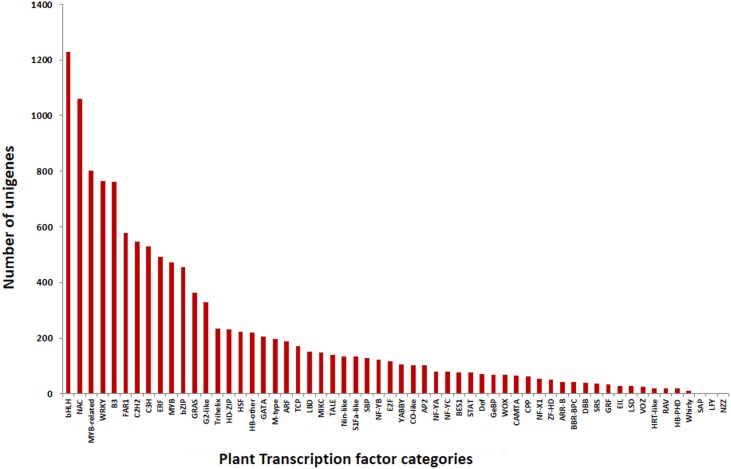
Summary of 12,539 unigenes of *M*. *Indica* classified into 58 Transcription Factor (TF) category. Among them bHLH, NAC, MYB, WRKY proteins were the most abundant.

**Fig 4:**
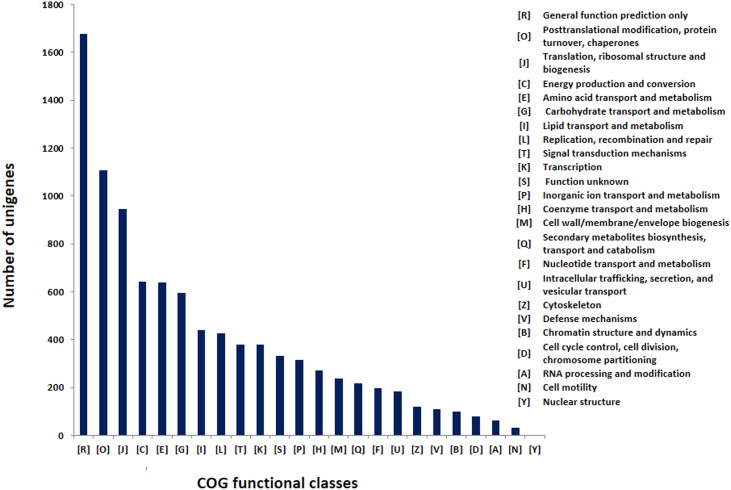
COG functional classification. A total of 9,847 unigenes were assigned to 24 COG categories.

KASS server was used for pathway mapping and orthologous gene assignment for the assembled unigenes, and we mapped 4,977 unigenes to 349 different KEGG pathways. The most represented pathway in terms of total number of hits from the transcript data were “ribosome”, “biosynthesis of amino acids”, “carbon metabolism”, “spliceosome”, “purine metabolism” and RNA transport which is quite similar to the results with the transcriptome of mango variety ‘Kent’ which showed the maximum transcripts representations for “biosynthesis of amino acids”, “ribosome” and “RNA transport” [55], but it was different from the results with variety ‘Zill’ [53], where the maximum representation of transcripts was for “metabolic pathways”, “biosynthesis of secondary metabolites”, and “plant-pathogen interaction” pathways (S1 Table). Unigenes belonging to different transcription factor families were identified using local similarity search (BLASTx) against plant transcription factor database (PlantTFDB v3.0) and NCBI COG database. Total 12,539 and 9,847 unigenes showed significant similarities with the PlantTFDB and COG database, respectively and were categorized in to 58 PlantTFDB and 24 COG families. There are three important transcription factor families in plants, namely bHLH, NAC and MYB that have been studied in detail, and here out of the 12,539 significant matches the maximum number of unigenes were categorized in bHLH (1,229) followed by NAC (1,059), MYB (801), WRKY (766) and B3 (763) families of transcription factors (Fig 3). The COG classification of unigenes into different functional cluster of orthologous groups (COG) based on BLASTx search classified unigenes into 24 COG categories. The largest category was of general functions (1676), followed by post-translational modifications, protein turnover, chaperones (1106), translation, ribosomal structure and biogenesis (946), energy production and conversion (643), amino acid transport and metabolism (639) and carbohydrate transport and metabolism (596). The least represented categories were for cell motility (33) and nuclear structure (3), while no unigene was categorized into extracellular structures (Fig 4).

### Development and validation of genic-SSR markers

A total of 5,465 SSR loci of different categories (mono, di, tri, tetra, penta, hexa and complex) were identified in 4,912 contigs, representing 7.01% of the total 70,057 unigenes. We excluded mononucleotide repeats, complex SSR and those having total lengths of <10 bp because SSR marker based mono-nucleotide repeats are not reproducible due to recombination slippage and PCR amplification problems, whereas complex SSRs show the least polymorphism [39]. This exclusion left only 1,481 Type I SSRs for primer design. Among the SSR containing unigenes, 1,438 (95.52%) unigenes possessed a single SSR locus, while 43 (4.44%) unigenes had two or more SSR loci each. As expected for coding sequences, tri-nucleotides were the most common repeat units representing 774 (52.26%) of the total filtered SSR, followed by di-nucleotide 641 (43.28%), tetra-nucleotide 47 (3.17%), penta-nucleotide 12 (0.82%) and hexa-nucleotide 7 (0.47%) repeats. Maximum percentage of SSR repeats constituted by tri-nucleotide and di-nucleotide 985 (71.80%) while only 387 (28.20%) of SSR constituted of tetra-nucleotide, penta-nucleotide and hexa-nucleotide repeats. The most abundant SSRs were with five reiterations, the frequency of a given SSR structure and the number of repeat units in it showed an inverse relationship (S2 Fig). Hence, SSR loci with less than five repeats are expected to be even more abundant but were not included in the present investigation because they would be less useful in the study of detectable polymorphism [39]. SSR motifs showing more than ten reiterations were rare with a frequency range of 1.28%–0.06%. Total 133 distinct repeat motifs were identified in 1,438 genic-SSRs, the 11 most frequent motifs are shown in (S2 Table). Di-nucleotide repeats AG/CT, AT/AT and AC/GT were the most abundant SSR with frequencies of 19.04%, 17.95% and 6.65%, respectively. Among the tri-nucleotide repeats, AAG/CTT and ATC/GAT were the most abundant with frequencies of 17.75% and 8.33%, respectively.

**Fig 5:**
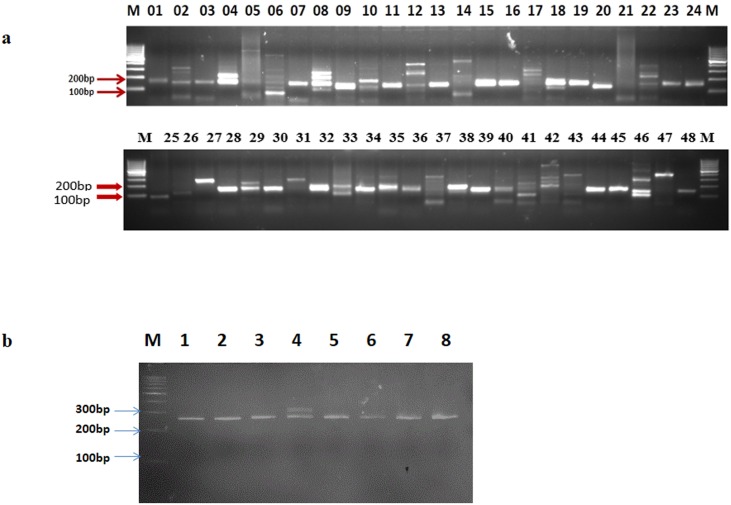
Wet lab validation of *in silico* designed genic-SSR markers of mango. **a)** PCR results with 48 different SSR markers (MSSR1- MSSR 48) in mango variety Amrapali; **b)** Allelic polymorphism of MSSR-13 in 8 different varieties of Mango 1. Neelam, 2. Dashehari, 3. Amrapali, 4. Chausa, 5. Pusa Lalima, 6. Ratual, 7. Mallika and 8. Alphonso.

PCR primers were designed successfully from the unique sequences flanking 1,069 SSR loci for the development of genic-SSR markers and were designated MSSR1 to MSSR1069 (M = Mango). Primers could not be designed for the remaining 4,396 SSR loci because their flanking sequences were either too short or the nature of sequence did not fulfill our criteria for primer design. Of the 1,069 SSR markers, 227 type I SSR loci (n ≥ 20 bp) (S3 Table) were filtered out and of this primers were synthesized for 100 loci for validation due to their high chance of showing polymorphism in agarose gel electrophoresis [56]. Out of the 100 synthesized primer pairs, 43 yielded PCR amplicon of expected size and were designated as “validated genic-SSR markers” (Table 2, Fig 5a). In addition, 36 primer pairs amplified ≥3 bands and 21 primer pairs failed to amplify even when the annealing temperature was reduced by 7°C (Fig 5b). All the amplified genic-SSR markers were scored for their amplicon size in eight varieties. Although a large proportion of the SSR loci were monomorphic, some of these will show polymorphism on analysis of a larger set of varieties. These markers have already been utilized successfully for diversity analysis in among 96 mango cultivars in a separate study [57]. Further, use of more sensitive techniques for DNA fragment size analysis, e.g. polyacrylamide gel electrophoresis or capillary electrophoresis, is also expected to show a higher rate of polymorphism.

**Table 2:** Details of 43 validated type I genic-SSR markers tested for polymorphism among 8 mango varieties.

S. No.	Marker Id.	(SSR motif) n	Product size	No. of alleles
1	MSSR4	(TAAAA)6	156	1
2	MSSR13	(ATC)9	153	2
3	MSSR15	(GGAACA)5	151	2
4	MSSR18	(GAC)7	161	1
5	MSSR22	(AAT)8	153	1
6	MSSR23	(TTA)7	150	1
7	MSSR31	(TTGT)5	151	1
8	MSSR37	(CCG)7	156	1
9	MSSR39	(TCA)8	172	1
10	MSSR40	(CA)10	159	1
11	MSSR48	(TATG)5	150	1
12	MSSR50	(TG)11	147	1
13	MSSR51	(TTC)7	150	2
14	MSSR54	(GTCAA)12	169	1
15	MSSR56	(CTCCAT)6	131	1
16	MSSR58	(TTTC)6	141	1
17	MSSR60	(AATA)5	150	1
18	MSSR62	(AAC)7	156	1
19	MSSR63	(GTT)8	152	1
20	MSSR66	(ATGG)6	156	1
21	MSSR67	(GCA)7	136	1
22	MSSR70	(ACCCT)5	154	1
23	MSSR71	(GAT)11	158	1
24	MSSR72	(CCA)7	183	1
25	MSSR73	(TACAG)12	144	1
26	MSSR74	(CCA)7	172	1
27	MSSR75	(TGTA)5	152	1
28	MSSR76	(GGTGG)5	161	2
29	MSSR77	(CAC)7	150	1
30	MSSR78	(TG)10	129	1
31	MSSR79	(ATG)7	158	1
32	MSSR80	(TC)11	152	2
33	MSSR81	(GTG)7	153	1
34	MSSR82	(AC)11	169	2
35	MSSR83	(CT)10	155	1
36	MSSR85	(AGG)7	177	1
37	MSSR86	(TTGT)5	150	1
38	MSSR91	(GCT)7	147	1
39	MSSR92	(ATCT)5	150	1
40	MSSR94	(CT)14	154	1
41	MSSR95	(AAC)7	142	1
42	MSSR96	(CT)11	170	1
43	MSSR100	(GGC)11	129	2

### SNP heterozygosity in mango varieties Neelam, Dashehari and their hybrid Amrapali

**Fig 6:**
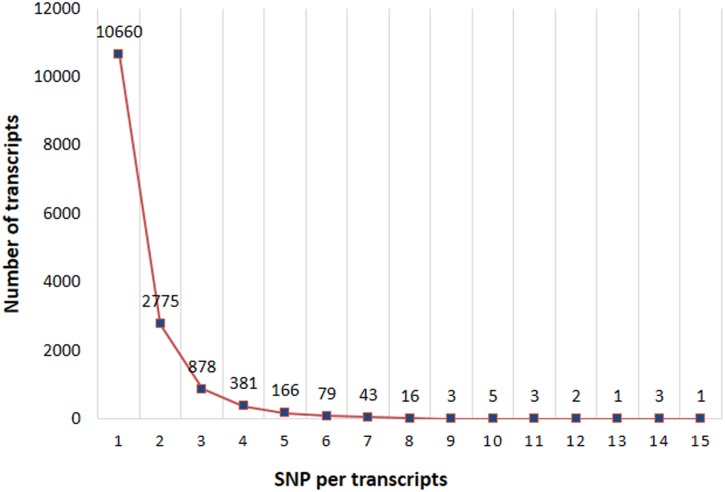
Frequency distribution of number of SNPs in mango leaf transcripts. Single SNP per transcripts were most abundant.

The non-redundant set of 70,057 transcripts was used as a reference for mapping of high quality reads from parents (‘Neelam’ and ‘Dashehari’) and hybrid (‘Amrapali’) and total 42,984 SNP positions were identified with full details saved as text file. In-house shell scripts was written which reads the transcript name and its SNP position from this text file, and extracts all the mapping reads information at each SNP position, including the full read name, base call in the mapped read at SNP position and position of the SNP in the reads as well as in the reference transcript. The results were tabulated in an Excel sheet, which included variety wise information on heterozygosity at each SNP position. This helped identify level of heterozygosity in the parents and hybrid ‘Amrapali’ using stringent criteria. After quality filtration we identified 22,306 SNPs in 10,571 transcripts common to all the three genotypes with an average of 2.1 SNPs and a range of 1–16 SNPs per contig (Fig 6).

We classified the SNPs into two categories: i) homozygous within a variety and ii) heterozygous within a variety, and found that in ‘Neelam’ the proportion of heterozygous SNPs was 49.3% while in ‘Dashehari’ it was only 30.19%. Interestingly, in their hybrid Amrapali the heterozygosity level increased to 64.5% (Table 3). Further, the 22,306 SNPs were classified in to eight categories on the basis of polymorphism in the hybrid ‘Amrapali’ *vis-a-vis* in its parental lines ‘Neelam’ and ‘Dashehari’ at the same position. ‘Amrapali’ showed 6,831 novel heterozygous SNP loci that were homozygous for contrasting alleles in the two parental varieties, which could be the basis of its superior performance. In addition, ‘Amrapali’ has maintained heterozygosity at 7,561 SNP loci that are heterozygous either in ‘Neelam’ (4,158 loci) or ‘Dashehari’ (2,049 loci) or both the parents (1,354 loci). On the other hand there were 6,760 SNP loci, which were heterozygous in either one or both the parents but became homozygous in ‘Amrapali’ (Table 3). The loss of heterozygosity from ‘Neelam’ was more than twice as compared to ‘Dashehari’, which is consistent with the higher level of heterozygosity in ‘Neelam’. Surprisingly, there were 2,061 SNP loci that were homozygous for contrasting alleles in the two parents but showed homozygosity in ‘Amrapali’, this was clearly due to lack of sufficient number of sequence reads from ‘Amrapali’ at these positions.

**Table 3:** Categorization of 22,306 SNPs into eight classes on the basis of heterozygosity in the hybrid Amrapali *vis a vis* its parental varieties Neelam and Dashehari.

Class	SNP Zygosity			No. of SNPs
	Neelam	Dashehari	Amrapali	
1	HM	HM	HT	6,831
2	HT	HM	HT	4,158
3	HM	HT	HT	2,049
4	HT	HT	HT	1,354
5	HT	HT	HM	2,061
6	HM	HM	HM	1,154
7	HM	HT	HM	1,270
8	HT	HM	HM	3,429
Total	11,002 HT	6,734 HT	14,392 HT	**22,306**

(HT = Heterozygous; HM = Homozygous).

## Conclusions

In this study we have presented development and validation of a comprehensive set of genic-SSR and SNP markers in mango by deep sequencing of leaf transcriptome using next generation sequencing technology. Considering the need to generate large number of molecular markers for mango breeding applications, a set of 288 type I genic-SSR markers were developed, and a subset of these was validated successfully for robust amplification in eight mango varieties. These markers can be used for diversity analysis and genetic mapping of useful horticultural traits in mango. In addition, an analysis of 22,306 SNP loci in hybrid mango clone ‘Amrapali’ and its parental lines ‘Neelam’ and ‘Dashehari’ revealed substantially higher heterozygosity in ‘Amrapali’. Among the parental lines ‘Neelam’ showed significantly higher level of heterozygosity than ‘Dashehari’. The identified genic-SNPs provide much-needed resource for the development of high density, cost-effective genotyping assays which would greatly help the mango breeding programs and genome wide association studies for the yield and quality traits in mango.

## Supporting Information

S1 Fig Plant species showing top BLASTx hit with mango transcripts. Mango transcripts showed highest similarity with *Citrus sinensis* (28.76%), *Citrus clementina* (17.43%), *Theobroma cacao* (6.8%), *Jatropha curcas* (4.44%) and *Vitis vinifera* (4.19%).
(TIF)

S2 Fig The frequency of SSR structure and the number of repeat units. The most abundant SSRs were with five reiterations, the frequency of a given SSR structure and the number of repeat units in it showed an inverse relationship.
(TIF)

S1 Table List of transcripts with KEGG Pathway Id and their categorization into various functional classes. (XLSX)

S2 Table Frequency distribution of SSR loci with different repeat motifs and number of repeats in the unigenes. (DOCX)

S3 Table List of genic-SSR markers along with their flanking primer sequences developed from mango leaf transcriptome unigenes. (XLSX)

## References

[1] Mukherjee SK. Cytological investigation of the mango (*Mangifera indica* L.) and the allied Indian species. Proceedings of National Institute of Science of India, New Delhi, 1950; 16:287–303.

[2] Arumuganathan K, Earle ED. Nuclear DNA content of some important plant species. Plant Mol. Biol. 1991; Rep. 9: 208–218. 10.1007/BF02672016

[3] FAOSTAT. Available online: http://faostat.fao.org/ (accessed on 15 November 2013).

[4] Tharanathan RN, Yashoda HM, Prabha TN. Mango (*Mangifera indica* L.), “The King of Fruits”—An Overview. Food Rev. Int. 2006;22:37–41.

[5] Singh NK, Mahato AK, Sharma N, Gaikwad K, Srivastava M, Tiwari K, et al. A draft genome of the king of fruit, mango (Mangifera indica L.). Plant and Animal Genome XXII Conference. 2014.

[6] Singh NK, Mahato AK, Jayaswal PK, Singh A, Singh S, Singh N, et al Origin, Diversity and Genome Sequence of Mango (Mangifera indica L.) Indian Journal of History of Science. 2016; 51.2.2:355–368.

[7] Mahattanatawee K, Manthey JA, Luzio G, Talcott ST, Goodner K, Baldwin EA. Total antioxidant activity and fiber content of select Florida-grown tropical fruits. J. Agric. Food Chem. 2006; 54 (19): 7355–63. 10.1021/jf060566s 16968105

[8] Rocha Ribeiro SM, Queiroz JH, Lopes Ribeiro de Queiroz ME, Campos FM, Pinheiro Santana HM. Antioxidant in mango (*Mangifera indica* L.) pulp. Plant Foods Hum Nutr. 2007; 62 (1): 13–7. 10.1007/s11130-006-0035-3 17243011

[9] Schnell RJ, Knight RJ. Frequency of zygotic seedlings from five polyembryonic mango rootstocks. Hort. Sci. 1992;27: 174–6.

[10] Truscott M. Biochemical screening of polyploid mango seedlings. CSFRI Information Bulletin. 1992;237: 17–8.

[11] Degani C, Cohen M, El-Batsri R, Gazit S. PGI isozyme diversity and its genetic control in mango. Hort. Sci. 1992;27: 252–4.

[12] Degani C, Cohen M, Reuvani O, El-Batsri R, Gazit S. Frequency and characteristic of zygotic seedlings from polyembryonic mango cultivars determined using isozymes as genetic markers. Acta Horticulturae. 1993;341: 78–85. 10.17660/ActaHortic.1993.341.6

[13] Hirano R, Htun Oo T, Watanabe KN. Myanmar mango landraces reveal genetic uniqueness over common cultivars from Florida, India, and Southeast Asia. Genome. 2010; 53(4):321 10.1139/g10-005 20616863

[14] Khan IA, Azim MK. Variations in intergenic spacer rpl20- rps12 of Mango (*Mangifera indica*) chloroplast DNA: implications in cultivar identification. Plant Evol Syst. 2011; 292(3–4):249–255. 10.1007/s00606-011-0424-4

[15] Ravishankar KV, Mani BH, Anand L, Dinesh MR. Development of new microsatellite markers from mango (Mangifera *indica*) and cross-species amplification. American Journal of Botany. 2011;98: e 96 –e99. 10.3732/ajb.1000263 21613158

[16] Souza IG, Valente SE, Britto FB, de Souza VA, Lima PS. RAPD analysis of the genetic diversity of mango (*Mangifera indica*) germplasm in Brazil. Genet. Mol. Res. 2011; 10(4):3080–3089. 10.4238/2011.December.14.1 22194163

[17] Srivastava N, Bajpai A, Chandra R, Rajan S, Muthukumar M, Srivastava MK. Comparison of PCR based marker systems for genetic analysis in different cultivars of mango. J. Environ. Biol. 2012; 33(2):159–166. 23033674

[18] Buschiazzo E, Gemmell NJ. The rise, fall and renaissance of microsatellites in eukaryotic genomes. BioEssays. 2006;28: 1040–1050. 10.1002/bies.20470 16998838

[19] Kelkar YD, Tyekucheva S, Chiaromonte F, Makova KD. The genome-wide determinants of human and chimpanzee microsatellite evolution. Genome Research. 2008;18: 30–38. 10.1101/gr.7113408 18032720 PMC2134767

[20] Csencsics D, Rodbeck SB, Holderegger R. Cost-effective, species-specific microsatellite development for the endangered dwarf bulrush (Typha *minima*) using next-generation sequencing technology. Journal of Heredity. 2010;101: 789–793. 10.1093/jhered/esq069 20562212

[21] Dutta S, Kumawat G, Singh BP, Gupta DK, Singh S, Dogra V, et al Development of genic-SSR markers by deep transcriptome sequencing in pigeonpea [Cajanus *cajan* (L.) Millspaugh]. BMC Plant Biology. 2011;11: 17–29. 10.1186/1471-2229-11-17 21251263 PMC3036606

[22] Van Deynze A, Stoffel K, Buell CR, Kozik A, Liu J, van der Knaap E, et al Diversity in conserved genes in tomato. BMC Genomics. 2007; 8:465 10.1186/1471-2164-8-465 18088428 PMC2249608

[23] Van Deynze A, Stoffel K, Lee M, Wilkins TA, Kozik A, Cantrell RG, et al Sampling nucleotide diversity in cotton. BMC Plant Biol. 2009; 9:125 10.1186/1471-2229-9-125 19840401 PMC2771027

[24] Ashrafi H, Hill T, Stoffel K, Kozik A, Yao JQ, Chin-Wo SR, et al De novo assembly of the pepper Transcriptome (*Capsicum annuum*): A benchmark for in silico discovery of SNPs, SSRs and candidate genes. BMC Genomics. 2012; 13:571 10.1186/1471-2164-13-571 23110314 PMC3545863

[25] Edwards’s D, Batley J. Plant genome sequencing: applications for crop improvement. Plant Biotechnol. J. 2010;8: 2–9. 10.1111/j.1467-7652.2009.00459.x 19906089

[26] Zalapa JE, Cuevas H, Zhu H, Steffan S, Senalik D, Zeldin E, et al Using next-generation sequencing approaches to isolate simple sequence repeat (SSR) loci in the plant sciences. American Journal of Botany. 2012; 99 (2): 193–208. 10.3732/ajb.1100394 22186186

[27] Ekblom R, Galindo J. Applications of next generation sequencing in molecular ecology of non-model organisms. Heredity.2011; 107: 1–15. 10.1038/hdy.2010.152 21139633 PMC3186121

[28] Li YC, Korol AB, Fahima T, Beiles A, Nevo E. Microsatellites: Genomic distribution, putative functions, and mutational mechanisms: A review. Molecular Ecology. 2002;11: 2453–2465. 10.1046/j.1365-294X.2002.01643.x 12453231

[29] Kircher M, Kelso J. High-throughput DNA sequencing—concepts and limitations. Bioessays. 2010; 32(6):524–36. 10.1002/bies.200900181 20486139

[30] Barabaschi D, Guerra D, Lacrima K, Laino P, Michelotti V, Urso S, et al Emerging knowledge from genome sequencing of crop species. *Mol*. Biotechnol. 2012;50: 250–266. 10.1007/s12033-011-9443-1 21822975

[31] Egan AN, Schlueter J, Spooner DM. Applications of next-generation sequencing in plant biology. Amer. J. Bot. 2012;99: 175–185. 10.3732/ajb.1200020 22312116

[32] Mardis ER. The impact of next-generation sequencing technology on genetics. Trends Genet. 2008;24:133–141. 10.1016/j.tig.2007.12.007 18262675 PMC2680276

[33] Zerbino D, Birney E. Velvet: algorithms for de novo short read assembly using de Bruijn graphs. Genome Res. 2008; 18:821–829. 10.1101/gr.074492.107 18349386 PMC2336801

[34] Schulz MH, Zerbino DR, Vingron M, Birney E. Oases: robust de novo RNA-seq assembly across the dynamic range of expression levels. Bioinformatics. 2012; 4 15; 28(8):1086–92. 10.1093/bioinformatics/bts094 22368243 PMC3324515

[35] http://www.clcbio.com.

[36] Huang X, Madan A. CAP3: A DNA sequence assembly program. Genome Res. 1999; 9;9(9):86 10.1101/gr.9.9.868 10508846 PMC310812

[37] http://pgrc.ipk-gatersleben.de/misa/ (15 June 2012, date last accessed).

[38] You FM, Huo N, Gu YQ, Luo MC, Ma Y, Hane D, et al BatchPrimer3: A high throughput web application for PCR and sequencing primer design. BMC Bioinformatics. 2008; 9:253 10.1186/1471-2105-9-253 18510760 PMC2438325

[39] Dutta S, Mahato AK, Sharma P, Raje RS, Sharma TR, Singh NK. Highly variable ‘Arhar’ simple sequence repeat markers for molecular diversity and phylogenetic studies in pigeonpea [Cajanus cajan (L.) Millisp.] Plant Breeding. 2012;132(2):1439–0523.

[40] Doyle JJ, DOYLE JL. Isolation of plant DNA from fresh tissue. Focus (San Francisco, Calif.) 1990; 12: 13–15.

[41] Altschul SF, Gish W, Miller W, Myers EW, Lipman DJ. Basic local alignment search tool. J. Mol. Biol. 1990; 215:403–410. 10.1016/S0022-2836(05)80360-2 2231712

[42] Altschul SF, Madden TL, Schaffer AA, Zhang J, Zhang Z, Miller W, et al Gapped BLAST and PSI-BLAST: a new generation of protein database search programs. Nucleic Acids Res. 1997; 25:3389–3402. 10.1093/nar/25.17.3389 9254694 PMC146917

[43] Conesa A, Getz S, Garcia-Gomez JM, Terol J, Talón M, Robles M. Blast2GO: a universal tool for annotation, visualization and analysis in functional genomics research. Bioinformatics. 2005; 21(18):3674–3676. 10.1093/bioinformatics/bti610 16081474

[44] Ye J, Fang L, Zheng H, Zhang Y, Chen J, Zhang Z, et al WEGO: a web tool for plotting GO annotations. Nucleic Acids Res. 2006; 34: W293–W297. 10.1093/nar/gkl031 16845012 PMC1538768

[45] Jin J, Zhang H, Kong L, Gao G, Luo J. PlantTFDB 3.0: a portal for the functional and evolutionary study of plant transcription factors. Nucleic Acids Res. 2014; 42: D1182–1187. 10.1093/nar/gkt1016 24174544 PMC3965000

[46] Tatusov RL, Fedorova ND, Jackson JD, Jacobs AR, Kiryutin B, Koonin EV, et al The COG database: an updated version includes eukaryotes. BMC Bioinformatics. 2003; 4(1):41 10.1186/1471-2105-4-41 12969510 PMC222959

[47] Moriya Y, Itoh M, Okuda S, Yoshizawa AC, Kanehisa M. KAAS: an automatic genome annotation and pathway reconstruction server. Nucleic Acids Research. 2007; 35(Web Server issue):W182–W185. 10.1093/nar/gkm321 17526522 PMC1933193

[48] Kanehisa M, Goto S. KEGG: Kyoto Encyclopedia of Genes and Genomes. Nucleic Acids Res. 2000; 28, 27–30. 10.1093/nar/28.1.27 10592173 PMC102409

[49] Li H, Durbin R. Fast and accurate short read alignment with Burrows—Wheeler transform. Bioinformatics. 2009; 25(14):1754–1760. 10.1093/bioinformatics/btp324 19451168 PMC2705234

[50] Li H, Handsaker B, Wysoker A, Fennell T, Ruan J, Homer N, et al The Sequence Alignment/Map format and SAMtools. Bioinformatics. 2009; 25: 2078–2079. 10.1093/bioinformatics/btp352 19505943 PMC2723002

[51] Koboldt DC, Zhang Q, Larson DE, Shen D, McLellan MD, Lin L, et al VarScan 2: Somatic mutation and copy number alteration discovery in cancer by exome sequencing. Genome Research. 2012; 22: 568–576. 10.1101/gr.129684.111 22300766 PMC3290792

[52] Azim KM, Khan IA, Zhang Y. Characterization of mango (*Mangifera indica* L.) Transcriptome and chloroplast genome. Plant Mol Biol. 2014; 85:193–208 10.1007/s11103-014-0179-8 24515595

[53] Wu H, Jia H, Ma X, Wang S, Yao Q, Xu W, et al Transcriptome and proteomic analysis of mango (*Mangifera indica* Linn) fruits Journal of proteomics. 2014; 10.1016/j.jprot.2014.03.030. 10.1016/j.jprot.2014.03.030 24704857

[54] Luria N, Sela N, Yaari M, Feygenberg O, Kobiler I, Lers A, et al De-novo assembly of mango fruit peel transcriptome reveals mechanisms of mango response to hot water treatment. BMC Genomics. 2014; 15:957 10.1186/1471-2164-15-957 25373421 PMC4236434

[55] Mitzuko DC, Adrian OL, Carmen Arminda CV, Magda Adelina PS, Sergio CF, Alejandro SF, et al Mango (*Mangifera indica* L.) cv. Kent fruit mesocarp de novo transcriptome assembly identifies gene families important for ripening. Frontiers in Plant Science. 2015; Vol-6 10.3389/fpls.2015.00062 25741352 PMC4332321

[56] Singh H, Deshmukh RK, Gaikwad K, Sharma TR, Mohapatra T, Singh NK. Highly variable SSR markers suitable for rice genotyping using agarose gels. Mol Breeding. 2010; 25:359–364. 10.1007/s11032-009-9328-1

[57] Lal S. Identification of genomic regions for alternate bearing and fruit quality traits in mango (Mangifer indica L.). 2016; Ph.D. thesis. Fruits and Horticultural Technology, P.G. School, ICAR- Indian Agriculture research Institute, New Delhi, pp 1–93.

