# Supplementary figures and images for: Leaf Transcriptome Sequencing for Identifying Genic-SSR Markers and SNP Heterozygosity in Crossbred Mango Variety ‘Amrapali’ (Mangifera indica L.)
Source: PLoS One. 2016 Oct 13;11(10):e0164325. doi: 10.1371/journal.pone.0164325 (PMC5063295; doi:10.1371/journal.pone.0164325)

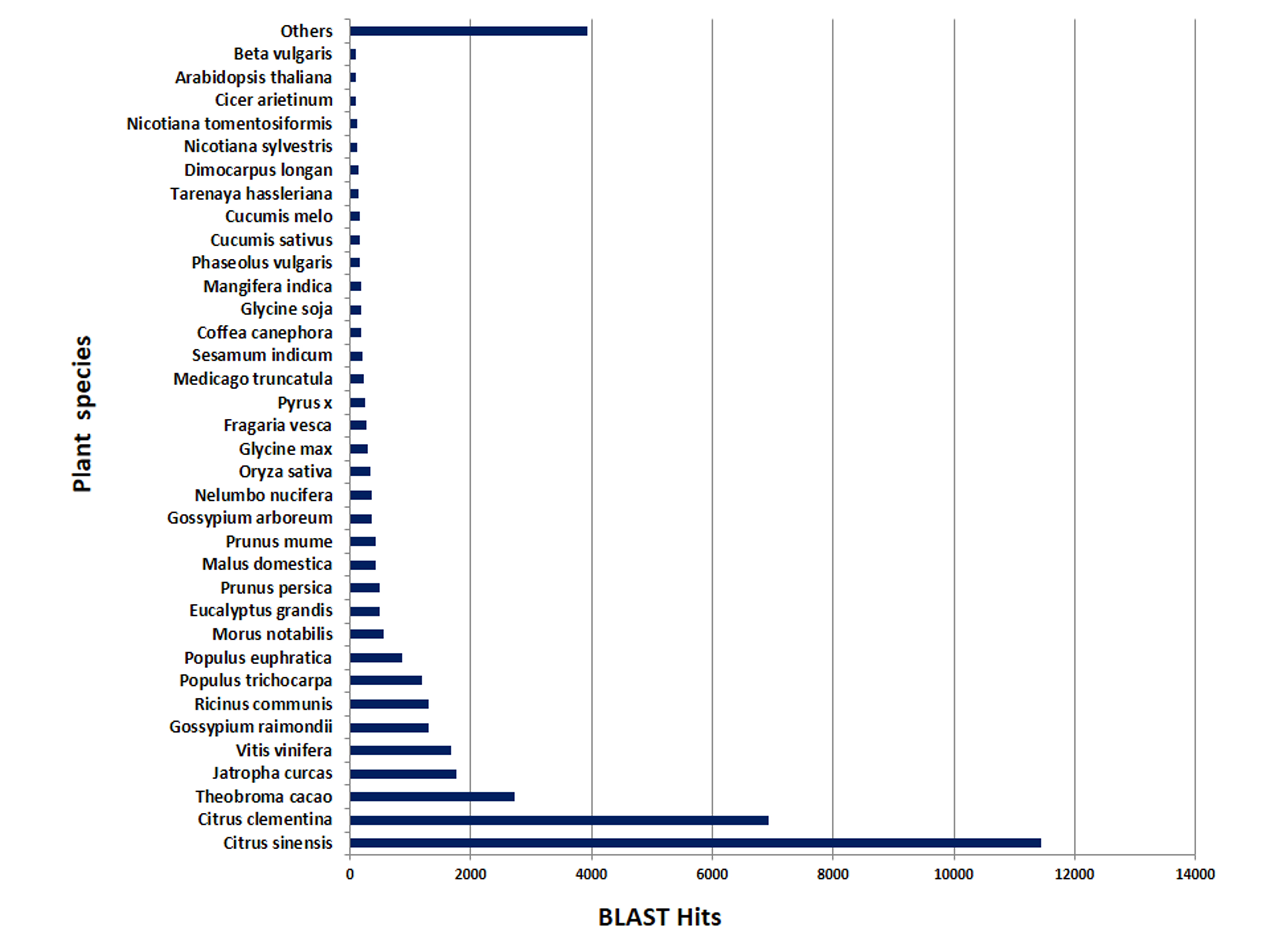

Supplement: S1 Fig — Mango transcripts showed highest similarity with Citrus sinensis (28.76%), Citrus clementina (17.43%), Theobroma cacao (6.8%), Jatropha curcas (4.44%) and Vitis vinifera (4.19%). (TIF) [file pone.0164325.s001.tif]

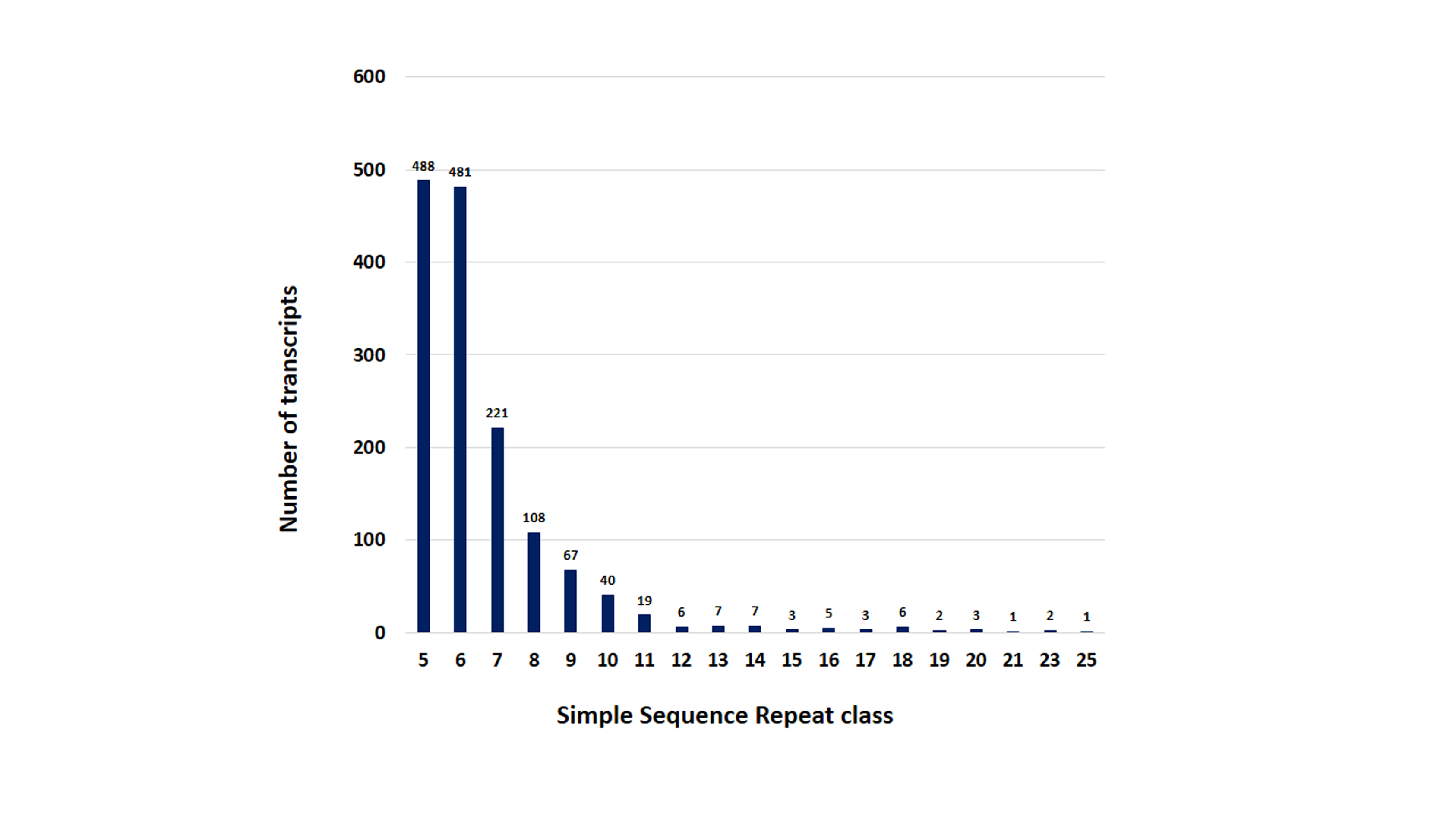

Supplement: S2 Fig — The most abundant SSRs were with five reiterations, the frequency of a given SSR structure and the number of repeat units in it showed an inverse relationship. (TIF) [file pone.0164325.s002.tif]
