# Supplementary material for: Leaf Transcriptome Sequencing for Identifying Genic-SSR Markers and SNP Heterozygosity in Crossbred Mango Variety ‘Amrapali’ (Mangifera indica L.)
Source: PLoS One. 2016 Oct 13;11(10):e0164325. doi: 10.1371/journal.pone.0164325 (PMC5063295; doi:10.1371/journal.pone.0164325)
Supplement: S2 Table — (DOCX) [file pone.0164325.s004.docx]

**Supplementary Table 2. Frequency distribution of the 11 most frequent SSR repeat motifs in the mango unigenes**

| **Number of reiteration of motif** | | | | | | | | | | | | | |
| --- | --- | --- | --- | --- | --- | --- | --- | --- | --- | --- | --- | --- | --- |
| **S.No.** | **Repeats** | **5** | **6** | **7** | **8** | **9** | **10** | **11** | **12** | **13** | **14** | **15** | **TOTAL** |
| **1** | AC/GT | 0 | 52 | 17 | 14 | 8 | 5 | 3 | 1 | 1 |  | 2 | 103 |
| **2** | AG/CT | 0 | 118 | 64 | 38 | 28 | 20 | 5 | 3 | 1 | 6 | 12 | 295 |
| **3** | AT/AT | 0 | 111 | 63 | 36 | 25 | 11 | 7 | 2 | 6 | 1 | 16 | 278 |
| **4** | CG/CG | 0 | 46 | 21 |  | 2 | 1 | 1 |  |  |  | 0 | 71 |
| **5** | AAC/GTT | 39 | 11 | 10 | 4 | 1 | 1 |  |  |  |  | 1 | 67 |
| **6** | AAG/CTT | 137 | 81 | 22 | 12 | 6 | 8 | 4 | 2 | 1 | 1 | 1 | 275 |
| **7** | AAT/ATT | 64 | 30 | 13 | 4 | 1 | 1 | 1 |  |  |  | 1 | 115 |
| **8** | ACC/GGT | 46 | 37 | 10 | 3 | 1 | 1 |  |  |  |  | 0 | 98 |
| **9** | AGC/CTG | 53 | 11 | 2 |  |  |  |  |  |  |  | 0 | 66 |
| **10** | AGG/CCT | 32 | 12 | 6 | 2 |  |  |  |  |  |  | 0 | 52 |
| **11** | ATC/ATG | 68 | 31 | 18 | 8 | 2 | 1 |  |  |  | 1 | 0 | 129 |
|  | **TOTAL** | **439** | **540** | **246** | **121** | **74** | **49** | **21** | **8** | **9** | **9** | **33** | **1549** |

- Additional 111 types of repeat motifs in the mango unigenes with frequency less than 52 each
